# Development of an Indirect ELISA to Detect African Swine Fever Virus pp62 Protein-Specific Antibodies

**DOI:** 10.3389/fvets.2021.798559

**Published:** 2022-01-13

**Authors:** Kexin Zhong, Mengmeng Zhu, Qichao Yuan, Zhibang Deng, Simeng Feng, Daoxin Liu, Xiaomin Yuan

**Affiliations:** ^1^Lab of Animal Disease Prevention and Control and Animal Model, Hunan Provincial Key Laboratory of Protein Engineering in Animal Vaccines, College of Veterinary Medicine, Hunan Agricultural University (HUNAU), Changsha, China; ^2^Animal Disease Prevention and Control Center, Changsha, China

**Keywords:** African swine fever virus, indirect ELISA, CP530R gene, pp62 protein, prokaryotic expression system

## Abstract

African swine fever (ASF) is a highly detrimental viral disease caused by African swine fever virus (ASFV). The occurrence and prevalence of this disease have become a serious threat to the global swine industry and national economies. At present, the detection volume of African swine fever is huge, more sensitive and accurate detection techniques are needed for the market. pp62 protein, as a protein in the late stage of infection, has strong antigenicity and a high corresponding antibody titer in infected pigs. In this study, the CP530R gene was cloned into expression vector pET-28a to construct a prokaryotic expression plasmid, which was induced by IPTG to express soluble pp62 protein. Western blot analysis showed that it had great reactivity. Using the purified recombinant protein as an antigen, an indirect ELISA method for detecting ASFV antibody was established. The method was specific only to ASFV-positive serum, 1:1600 diluted positive serum could still be detected, and the coefficients of variation (CV) of the intra assay and inter assay were both <10%. It turns out that the assays had excellent specificity, sensitivity, and repeatability. This provides an accurate, rapid, and economical method for the detection of ASFV antibody in clinical pig serum samples.

## Introduction

African swine fever (ASF) is a viral disease of swine that is caused by African swine fever virus (ASFV), and mainly leads to acute, fearful, and serious contact infectious disease in domestic pigs and wild boars (1). In view of the seriousness of ASF, the World Organization for Animal Health (OIE) has listed it as a notifiable animal disease, which is also a Class 1 animal infectious disease in China (2). Furthermore, diseased pigs, rehabilitated pigs, and recessively infected pigs are the main infectious sources of the disease, and Ornithodoros soft ticks carrying ASFV are also one of the infectious sources (3).

Since ASF was first reported in Kenya, Africa, in 1921, it has been found in 60 countries across Europe, Latin America, and Asia (4). It harms the development of the swine raising industry and causes enormous economic losses in the world (5). Similarly, after the first case of ASF was reported in China in August 2018, the disease had a wide outbreak and epidemics in various regions of China, bringing tremendous economic losses to China's swine industry (2). To date, an effective and safe commercial ASFV vaccine is not available (6). The available methods of disease control and prevention consist of early diagnosis, rapid elimination of infectious sources, and blocking transmission routes (7). However, the number of samples to be screened in the process of early diagnosis is very large, so the required detection technology needs to have high sensitivity and accuracy. Therefore, it is of extreme significance to study the diagnostic methods of this disease. So far, there have been several routine laboratory diagnostic techniques for detecting ASFV, including polymerase chain reaction (PCR), loop-mediated isothermal amplification (LAMP), fluorescence quantitative PCR, erythrocyte adsorption (HAD), colloidal gold rapid strip, enzyme-linked immunosorbent assay (ELISA), and other serological tests. Within them, ELISA is the most commonly used method for specific antibody detection (8).

ASFV is a large, enveloped, complex icosahedral double-stranded DNA arbovirus, the sole member of the family Asfarviridae, genus Asfivirus (9). Domestic pigs, wild boars, and Ornithodoros soft ticks are the hosts of ASFV, and the virus replicates mainly in the cytoplasm of porcine monocytes and macrophages (10, 11). The genes of this virus have a full length of 170–190 kb and encode more than 200 proteins (12). Thirty-eight proteins are known to be involved in nucleotide metabolism, gene transcription, viral replication, and repair functions (13). Among them, pp62 protein is a 63.5 kDa protein encoded by the CP530R gene. The protein sequence has high conservation and immunogenicity in different ASFV strains (14). Under the action of the S273R enzyme, the protein can be cleaved into mature viral proteins P35, P15, and P8 (15). P35 and P15 are involved in the formation of viral icosahedron and become momentous structural proteins. The mature products of pp62 are the basic components of the core and shell, and the mature products of pp220 together account for about 30% of the total protein of the virion (16). Thus, pp62 protein, as the precursor protein of structural proteins P35 and P15, may be one of the vital targets for the immunological diagnosis of African swine fever virus, and has immense significance in the basic research of the pathogenesis and immune mechanism of African swine fever virus.

In this study, we expressed and purified the ASFV pp62 protein, and established an indirect ELISA method for the detection of ASFV antibodies using pp62 protein as a diagnostic antigen, which laid the foundation for the development of ASFV early diagnosis kits and the structural and functional study of pp62 protein.

## Materials and Methods

### Serum Samples

Positive standard sera and negative standard sera were obtained from the Animal Disease Prevention and Control Center (Changsha, China). The negative serum samples (*N* = 40) were decided through RT-PCR and a commercial ELISA (ID. Vet Inc., France). Clinical Serum samples (*N* = 350) were collected from sows and adult pigs on commercial swine farms from 2018 to 2021.

### Cloning and Expression of pp62 Protein

The full length CP530R coding region of ASFV (GenBank accession, No.FR682468.2) was synthesized by Tsingke Biotechnology Co., Ltd. (Changsha, China) and amplified using F(5′CGGAATTCATGCCCTCTAATATGAAACAGTT) and R(5′CCAAGCTTTTATTCTTGAAGTAACTTTAGT) primers appended with *Eco*RI and *Hin*dIII restriction sites, respectively. A recombinant plasmid was constructed by ligating the PCR product into the vector pET-28a. The ligation product was transformed into *E. coli* DH5α competent cells, and was verified using double restriction enzyme digestion and sequencing. *E. coli* BL21 competent cells were transformed with recombinant plasmid, and protein expression was induced with 1 mM IPTG at 27°C for 9 h. Nickel-nitrilotriacetic acid (Ni-NTA) metal affinity chromatography was used to purify the recombinant pp62 protein, which was identified with SDS-PAGE and Western blot. ASFV-positive pig serum was used as the primary antibody (dilution of 1:3,000) for the Western blot assay.

### Checkerboard Titration

The antigen and antibody concentration were optimized using checkerboard titration. Briefly, the recombinant antigen was coated to the plate in concentrations of 0.5–2 μg/mL. Positive and negative standard sera were diluted at 1:50–1:400. Then HRP anti-swine IgG (SeraCare, USA) was diluted at 1:5,000–1:10,000 to determine the optimal conjugate dilution. The conditions that gave the highest OD450 ratio between the positive and negative sera (*P*/*N* value) and an OD450 value for positive serum close to 1.0 were scored as optimal working conditions.

### Cut-Off Value for pp62-IELISA

A total of 40 negative serum samples from uninfected pigs were used to calculate the cut-off value of the indirect ELISA. Statistical analysis was performed to calculate the mean value ($\bar{X}$) and standard deviation (SD) of the OD450 values. The cut-off value was determined as $\bar{X}$ + 3SD.

### Specificity and Sensitivity of pp62-IELISA

The established pp62-iELISA assay was used against respiratory syndrome virus (PRRSV), porcine circovirus type 2(PCV2), classical swine fever virus (CSFV), pseudorabies virus (PRV), foot-and-mouth disease virus (FMDV)-positive sera as well as ASFV-positive and negative sera. ASFV-positive serum was diluted from 1:100 to 1:6,400 to determine the highest dilution of serum. The sensitivity of the ELISA was evaluated based on the cut-off.

### Reproducibility of pp62-IELISA

Intra and inter-assay variation (coefficient of variation [CV]) between runs were evaluated. Briefly, five sera were randomly selected. Three replicates of each sample were assayed in one batch to evaluate intra-assay (within plate) variation and three plates were assayed as separate batches to evaluate inter-assay (between assays) variation.

### Comparison of pp62-IELISA With Commercial Kits

A total of 350 clinical serum samples were tested using pp62-iELISA. Results were compared with commercial kits (ID. Vet, France) to evaluate the performance of pp62-iELISA in terms of relative sensitivity [(true positive/(true positive + false negative)]^*^ 100% and relative specificity [(true negative/(true negative + false positive)]^*^ 100%.

### Statistical Analysis

All data were analyzed using the Prism 5 software (GradphPad Software, La Jolla, CA, USA). All data were analyzed using a two-tailed student's *t*-test. *P* < 0.05 was considered statistically significant.

## Results

### Expression and Purification of pp62 Protein

**Figure 1:**
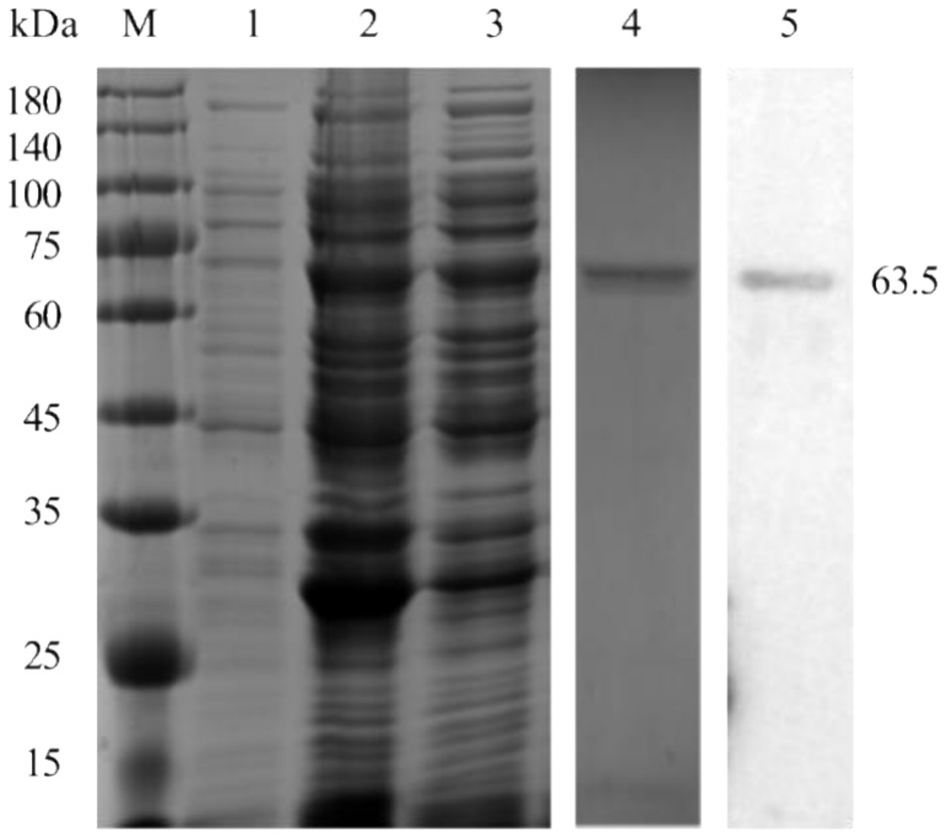
Expression and purification of the pp62 protein. M-Protein marker, 1-uninduced bacterial culture, 2-induced bacterial lysate, 3- soluble fraction, 4-SDS-PAGE analysis of purified pp62 protein, and 5-Western blotting analysis of purified pp62 protein.

pp62 protein (63.5 kDa) was successfully expressed in the soluble fraction and confirmed by Western blot analysis using ASFV-positive pig serum (Figure 1).

### Optimization of the Working Conditions of pp62-IELISA

Checkerboard titration was applied to investigate the concentration of the coating antigen and sera. The maximum *P*/*N* (6.08) value was obtained when the concentration of pp62 protein was 2 μg/mL and the dilution of serum was 1:400 (Table 1). Furthermore, other reaction conditions of the developed ELISA were optimized. In brief, the optimum coating condition was 2 h at 37°C. The best blocking solution was selected as 5% skimmed milk in PBST. The optimal reaction times for serum, secondary antibodies, and TMB solution were 45, 30, and 10 min, respectively. Finally, the best working dilution of the HRP anti-pig IgG was 1:7,500.

**Table 1:** Results of the *P*/*N* values at different conditions.

**Antigen at different concentration (μg/mL)**		**Dilution of sera**			
		**1:50**	**1:100**	**1:200**	**1:400**
	P	2.415	2.075	1.669	1.276
4	N	0.443	0.368	0.303	0.231
	P/N	5.45	5.63	5.51	5.52
	P	2.261	1.827	1.396	1.083
2	N	0.396	0.337	0.241	0.178
	P/N	5.71	5.42	5.79	6.08
	P	1.934	1.537	1.074	0.773
1	N	0.370	0.321	0.232	0.174
	P/N	5.23	4.78	4.62	4.44
	P	1.786	1.473	0.926	0.686
0.5	N	0.329	0.288	0.225	0.165
	P/N	5.43	5.11	4.12	4.16

### Repeatability of the pp62-IELISA

The intra-batch variation and inter-batch variation were used to validate the repeatability of the pp62-iELISA. The coefficients of variation (CV) of both the intra-assay and inter-assay were lower than 10% (Table 2). These results indicated that the pp62-iELISA has high repeatability and low variability.

**Table 2:** Results of the repeatability assay for pp62-iELISA.

**Sample number**	**Inter-assay CV (%)**		**Intra-assay CV (%)**	
	**X ± SD**	**CV (%)**	**X ± SD**	**CV (%)**
1	1.045 ± 0.010	0.96	0.999 ± 0.038	3.80
2	0.415 ± 0.019	4.58	0.440 ± 0.015	3.40
3	1.437 ± 0.023	1.60	1.452 ± 0.017	1.17
4	0.201 ± 0.003	1.49	0.236 ± 0.005	2.13
5	1.186 ± 0.024	2.02	1.214 ± 0.043	3.54

### Determination of Cut-Off Value

**Figure 2:**
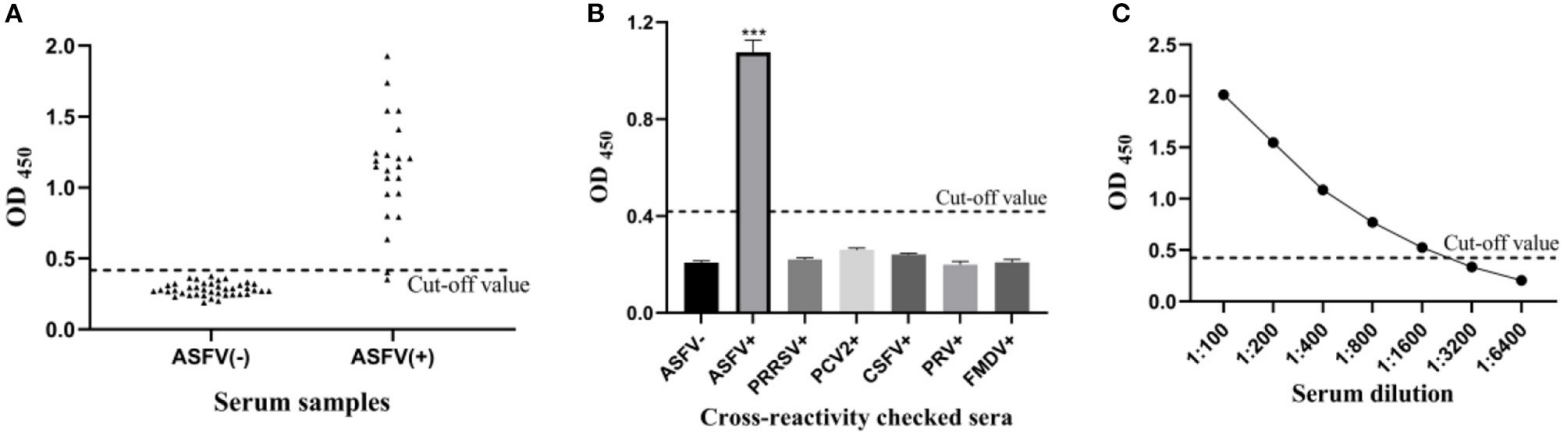
Sensitivity and specificity of the pp62-iELISA. **(A)** Determination of the cut-off value of pp62-iELISA (OD_450_ = 0.418). Distributions of OD values determined for ASFV-negative (*N* = 40) and ASFV-positive (*N* = 22) serum samples using pp62-iELISA; **(B)** the specificity test of pp62-iELISA. The pp62-iELISA detected no cross reactions with sera containing antibodies against five porcine pathogens; **(C)** sensitivity of the pp62-iELISA. ***p < 0.001.

The mean ($\bar{X}$) OD450 value of negative sera (*N* = 40) was 0.283, and the SD was 0.045, resulting in a cut-off value of 0.418 ($\bar{X}$ + 3SD = 0.418) (Figure 2A). Therefore, samples with OD450 values ≥0.418 were considered as positive, and samples with OD450 values <0.418 were considered negative.

### Diagnostic Sensitivity and Specificity of pp62-IELISA

The optimized indirect ELISA antibody detection method was used to test PRRSV, PCV2, CSFV, PRV, and FMDV antibody-positive pig serum, and the results showed that all pathogen-positive sera tested negative except for the ASFV-positive control (Figure 2B). The results show that the sensitivity of the ELISA method established in this study is 1:1600 (Figure 2C), indicating that the sensitivity of the method is excellent.

### Evaluation of pp62-IELISA in Comparison With Commercial Kits

The performance of pp62-iELISA in terms of relative sensitive, specificity, and accuracy were compared with commercial kits (ID. Vet, France). The results are shown in Table 3. The evaluation shows the good relative sensitivity and specificity of the tests, the pp62-iELISA had 90.90 and 98.78% sensitivity and specificity, respectively. The compliance rate was 98.28%.

**Table 3:** Results of pp62-iELISA in comparison with commercial kits.

		**ID. Vet**		**Total**
		**+**	**−**	
pp62-iELISA	+	20	4	24
	−	2	324	326
Total		22	328	350

*Relative sensitivity= 20/ 22= 90.91%.*

*Relative specificity= 324/ 328= 98.78%.*

*Compliance rate= 344/ 350= 98.29%*.

## Discussion

ASF is an acute, febrile, and highly lethal contagious viral disease of swine caused by ASFV (17). The disease has a short duration, high morbidity and mortality, and the highly virulent strain can cause mortality of up to 100% in domestic pigs and wild boars (18). The natural hosts of this virus include wild suids and arthropod vectors of the Ornithodoros genus. It is also the most serious infectious disease in the pig industry, causing significant economic losses to the swine raising industry and the pork market worldwide (19).

ASFV has a great number of genotypes, and its immune escape mechanism is complex and diverse, which leads to numerous difficulties in vaccine research (18). As there is no commercially available vaccine, molecular diagnostic methods and serological detection techniques are still considered as the main means of identifying infected animals and controlling ASF (20). What is more, the market urgently needs sensitive and accurate detection technology. Among them, ELISA serological diagnosis is not only a mature technology and stable detection method, but also has simple operation and low cost, so it is widely used in practice (21). There are three main ELISA kits from Ingenasa, Svanova, and ID. Vet, among which Ingenasa's blocking ELISA is the recommended serological test in Europe. However, Gallardo et al. compared the performance of ID. Vet's competition ELISA, Ingenasa's competition ELISA, and Svanova ELISA kits and indicated that the ID. Vet ELISA kit had a relatively low false positive rate and better performance (22). And in practical application, these three kits are expensive and stable supply cannot be guaranteed. With the emergence of mutated ASFV strains with weak virulence in various countries, nucleic acid fluorescence PCR combined with serum antibody detection is a common method to confirm diagnosis (23). Therefore, there is an urgent need to develop accurate serological antibody detection methods. Currently, serological diagnostic target proteins mainly focus on p72, CD2v, p54, p30, and pp62, which have strong antigenicity and high corresponding antibody titers in infected pigs (24). These proteins can serve as antigens to establish corresponding serological detection methods for antibody or antigen detection of ASFV.

pp62 protein is encoded by the ASFV CP530R gene with a molecular weight of 63.5 kDa (25). As a polymeric protein, pp62 protein is co-assembled with another polymeric protein, pp220, to form the inner core shell of ASFV (15). The mature products of pp62, p35, and p15 participate in the icosahedron formation of the virus and are the basic components of the core and shell. Gallardo et al. used the arbovirus expression system to express ASFV pp62 protein, and thus established an indirect ELISA (iELISA) method (26). Further research indicated that pp62-iELISA had better sensitivity and specificity than p30-iELISA and p54-iELISA, and could detect poorly preserved serum. Compared with the arbovirus expression system, the production cost of the prokaryotic expression system is relatively low, and the prepared protein is stable and easy to purify, which is suitable for large-scale production (27, 28). Therefore, ASFV pp62 protein was expressed by the prokaryotic expression system in this study. The results showed that the recombinant protein was soluble and had strong reactivity. The recombinant protein was used as an ELISA coating antigen to establish and optimize the indirect ELISA detection method.

The specificity study shows that the developed assay does not cross react with antibodies of other related swine viruses such as PRRSV, PCV2, CSFV, PRV, and FMDV. Sensitivity studies showed that the method could detect commercial positive serum of ASFV at a maximum dilution of 1:1,600. The results of the repeatability test showed that the CV in both intra-batch and inter-batch repeated tests was <10%, indicating that the pp62-iELISA established in this study had good repeatability. The coincidence rate with the ID. Vet ELISA kits reached 98.28%. It can be applied to the detection of ASF antibody in clinical serum samples. This will play an important foundation for the further development of ASF antibody detection kits, and is of great significance to the prevention, control, and eradication of ASF.

## Conclusion

The pp62-iELISA established in the present study was repeatable and specific for ASFV antibody detection, simple and economical to produce and perform, and time-saving. The present report may facilitate the development of a reliable tool for the large-scale detection of ASFV antibodies.

## Data Availability Statement

Publicly available datasets were analyzed in this study. This data can be found at: https://www.ncbi.nlm.nih.gov/nuccore/FR682468.2.

## Author Contributions

XY designed the study. KZ designed and prepared the tables and figures. ZD and DL made substantial, direct, and intellectual contributions to the work. KZ and MZ contributed to the writing of the paper. KZ, QY, and SF performed and collected data from the experiment and analyzed data. All authors approved the article for publication.

## Funding

This study was supported by the Key Project of Research and Development Plan of Hunan Province (2019NK2171), the Natural Science Foundation of Hunan Province (2020JJ4041 and 2021JJ30316), the Outstanding Youth Scientist Foundation of Hunan Province (19B253), and the Youth Fund Project of Hunan Science and Technology Department (2020JJ5248).

## Conflict of Interest

The authors declare that the research was conducted in the absence of any commercial or financial relationships that could be construed as a potential conflict of interest.

## Publisher's Note

All claims expressed in this article are solely those of the authors and do not necessarily represent those of their affiliated organizations, or those of the publisher, the editors and the reviewers. Any product that may be evaluated in this article, or claim that may be made by its manufacturer, is not guaranteed or endorsed by the publisher.
